# Cognitive Behavioral Therapy for Sexual Concerns During Perimenopause: A Four Session Study Protocol

**DOI:** 10.3389/fgwh.2021.744748

**Published:** 2021-10-06

**Authors:** Sheryl M. Green, Melissa Furtado

**Affiliations:** ^1^Department of Psychiatry and Behavioural Neurosciences, McMaster University, Hamilton, ON, Canada; ^2^Women's Health Concerns Clinic, St. Joseph's Healthcare Hamilton, Hamilton, ON, Canada; ^3^Department of Psychology, Neuroscience and Behaviour, McMaster University, Hamilton, ON, Canada

**Keywords:** perimenopause, sexual concerns, non-pharmacological management, cognitive behavioral therapy, psychological

## Abstract

**Background:** During the menopausal transition, women often experience physical (e.g., vasomotor symptoms) and emotional (e.g., anxiety and depression) difficulties that significantly impact functioning and overall quality of life. Although sexual concerns (e.g., decreased sexual desire, orgasm), are reported by up to 87% of peri- and post-menopausal women, and are associated with adverse impact on functioning and distress, treatment options that directly target this area are limited, and most often involve medication (e.g., hormone replacement). Effectiveness of these treatments is often defined as improvements in physical symptoms, however, associated psychological and emotional symptoms rarely, if at all, improve. Cognitive behavioral therapy (CBT) has been proposed as a low-risk treatment for menopausal symptoms with studies showing improvement in frequently reported symptoms (e.g., vasomotor symptoms, depression, anxiety, sleep). Sexual concerns, however, have either not been directly targeted at all in current CBT protocols, or the very few protocols that include sexual concerns, demonstrated modest gains in sexual desire.

**Methods:** This protocol paper outlines the development, design, and implementation of a newly developed CBT for sexual concerns trial during perimenopause (CBT-SC-Peri). Although sexual concerns are prevalent during both the peri- and post-menopausal periods, we will be evaluating the effectiveness of a CBT-SC protocol specifically for perimenopausal women as a means of early intervention. The clinical sample will comprise 82 women aged 40–60 years currently in perimenopause, as per the Stages of Reproductive Aging Workshop (STRAW) definition, and medication stable (if applicable). To ensure participants are experiencing clinically significant sexual concerns, a baseline cut-off score of 26 or lower on the Female Sexual Functioning Index will be utilized. Exclusion criteria include participants with psychotic disorders, or current substance and/or alcohol dependence, or severely depressed/suicidal. The CBT-SC-Peri is a weekly, four session treatment, lasting up to 90 min per session and includes psychoeducation and cognitive and behavioral strategies designed to challenge unhelpful beliefs and promote healthy sexual behaviors. As this is an individual CBT protocol, content will be tailored to address the specific problems relevant for each participant. Eligible women will be placed directly into treatment or on a 4-week waitlist and reassessed prior to starting treatment. The primary outcome (sexual satisfaction), as well as secondary outcomes (desire, arousal, relationship satisfaction, body image, vasomotor symptoms, depression, and anxiety) are assessed at baseline, post-waitlist (for those on waitlist), and post-treatment.

**Discussion:** To our knowledge, this will be the first study to investigate the efficacy of a CBT protocol (CBT-SC-Peri) specifically aimed at improving sexual concerns experienced during perimenopause. If effective, this form of treatment may not only be preferred by some, but necessary for others as consumer demand increases for non-pharmacological treatments for perimenopausal symptoms. Further, this protocol can be integrated into perimenopausal care and will be made available by dissemination to healthcare practitioners.

**Clinical Trial Registration:** Trial # NCT04922385 and Accessible at: https://clinicaltrials.gov/ct2/show/NCT04922385?term=NCT04922385anddraw=2andrank=1.

## Introduction

The menopausal transition is often accompanied by adverse physical (e.g., vasomotor symptoms, sleep difficulties, sexual concerns) and emotional (e.g., anxiety, depression) changes that significantly impact a woman's functioning and overall quality of life (1–4). Sexual concerns are reported by as many as 68–86.5% of menopausal women (5) and yet have received little attention in the clinical literature. Commonly reported sexual concerns include decreased sexual desire, reduced ability to have an orgasm, and pain during intercourse. These sexual concerns may be due to decreases in estrogen levels leading to vulvovaginal atrophy (6), decreased elasticity of the vaginal walls, and decreased vascularization and innervation of vulvovaginal tissue (6). These physical changes can result in genital dryness and irritation, impaired perception of touch and vibration, and often dyspareunia, all contributing to a reduction in sexual satisfaction (7). Further, sexual concerns have been found to contribute to poor self-image, lower sense of sexual attractiveness, and can have a negative impact on physical and emotional well-being (8). Consistent with the World Health Organization's (9) definition of sexual health, sexual concerns exhibited during the menopausal transition are not just physical, but also psychological and emotional in nature. Despite the prevalence of sexual concerns, their impact on functioning and associated distress, treatment options that directly target this area of functioning among women going through the menopausal transition are limited.

The most commonly prescribed treatment for sexual concerns during the menopausal transition is hormone therapy (HT), which generally includes a combination of estrogen- and progesterone-based compounds (10, 11). For some women, HT may be associated with health risks, including an increased risk for cardiovascular events and breast cancer after long term use (12–14). Testosterone treatments have also been reported to improve sexual concerns during menopause, however, these treatments have also been associated with adverse side effects [e.g., higher incidence of hair growth, vaginal bleeding, weight gain, increased LDL-cholesterol; (13, 15)]. Further, effectiveness of these treatments is typically defined as improvements in physical symptoms, such as vaginal dryness, atrophy, and pain during intercourse. Psychological and emotional symptoms associated with sexual concerns during the menopausal transition (e.g., self-image, desire) are rarely, if at all, improved with these treatments (16).

Psychological therapy, specifically Cognitive Behavioral Therapy (CBT), has been proposed as a low-risk treatment for menopausal symptoms with studies showing CBT as effective in reducing several common menopausal symptoms [e.g., vasomotor symptoms, depression, anxiety, and sleep difficulties; (17–19)]. Sexual concerns, however, have either not been directly targeted at all in current CBT protocols, or the two protocols that have included sessions on sexual concerns (19, 20), have only demonstrated modest gains in a single domain (e.g., sexual desire). Further, most CBT studies for menopause have included broad measures of menopausal symptoms (e.g., the Greene Climacteric Scale, Menopause Rating Scale), which do not allow us to determine the impact of CBT on important domains of sexual functioning and satisfaction specifically sexual desire, interest, and self-image. As a result, it is still not known whether CBT for menopausal symptoms can improve a wide array of sexual concerns.

Given the high rates and associated impairment of sexual concerns during perimenopause, we developed a CBT protocol specifically designed to target sexual concerns during perimenopause as a means of early intervention. Although decline in sexual function and satisfaction naturally decreases with age (13), this decline may also significantly reduce quality of life. As a result, we aim to address sexual concerns earlier in the menopausal transition with the goal of both improving sexual satisfaction, as well as reducing the impact on one's quality of life to improve future outcomes. The primary objective of this study is to evaluate the efficacy of this weekly, four-session, individual CBT for sexual concerns in perimenopause (CBT-SC-Peri) protocol. It is hypothesized that the CBT-SC-Peri protocol will significantly improve sexual satisfaction (primary outcome), desire, arousal, relationship satisfaction, body image, vasomotor symptoms, depression, and anxiety (secondary outcomes). To our knowledge, this will be the first study to investigate the efficacy of a CBT protocol (CBT-SC-Peri) specifically aimed at improving sexual concerns and associated distress experienced during perimenopause. As consumer demand increases for non-pharmacological treatments for perimenopausal symptoms, this form of treatment may not only be preferred by some, but necessary for others as hormonal treatments commonly have associated adverse effects and/or are contraindicated for some women.

## Methods

### Participants

#### Inclusion/Exclusion Criteria

The clinical sample for this study will comprise participants who meet the following inclusion criteria: (1) 40–60 years of age, who are either single or have a partner; (2) peri-menopausal and have had a menstrual period within the previous 12 months as per the STRAW definition (21), (3) cut-off score of 26 or lower on the Female Sexual Functioning Index, indicating sexual dysfunction (22), (4) medication stable (e.g., HT), with no changes in dosing for the previous 3 months (19), (5) no psychological treatment to address sexual dysfunction and/or sexual concerns within the previous 6 months; and (6) speak, read, and write in English to comprehend testing procedures and written materials in treatment. Participants will be excluded if: (1) they have any psychotic disorders, or current substance and/or alcohol dependence; and/or (2) are severely depressed/suicidal at time of the intake assessment as they would not be able to meaningfully participant in treatment.

#### Sample Size

Based on our data from our CBT for menopausal symptoms randomized controlled trial (19), as well as in previous studies (20) assessing the efficacy of CBT for sexual concerns in menopausal women, we estimate a medium effect size of 0.5 for our study. Using an ANCOVA power analysis to calculate sample size, and with a level of significance set at α = 0.05 and a study power set at 80%, 68 participants are required to conduct our study. Adjusting our sample for a 20% attrition rate, we plan on recruiting 82 participants.

#### Study Design

**Figure 1:**
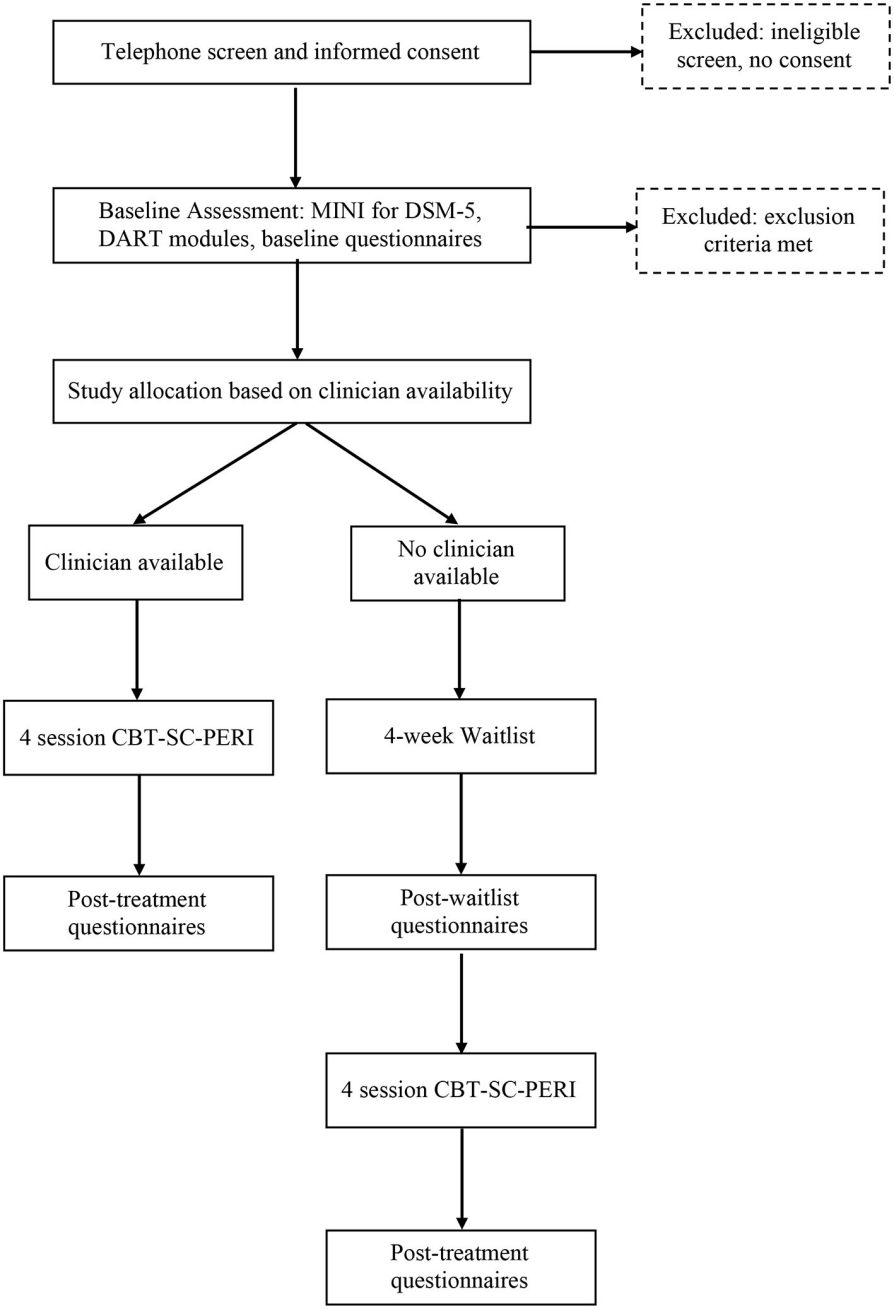
Flowchart of trial procedures.

This is an open waitlist study whereby eligible participants will be assigned to a treating clinician and will complete 4 weeks of the CBT-SC-Peri treatment protocol, followed by a post-treatment assessment. If a treating clinician is not immediately available, participants will be placed on that clinician's waitlist for 4 weeks. They will then be re-assessed after the 4-week waitlist period and then undergo the four-session treatment, followed by a post-treatment assessment. For a flowchart of trial procedures, see Figure 1.

### Procedure

#### Recruitment and Informed Consent

Participants will be recruited through the Women's Health Concerns Clinic (WHCC), St. Joseph's Healthcare (SJHH), a well-established clinical and research center dedicated to women's health issues, particularly those associated with reproductive life cycle events (e.g., perinatal, menopausal transition). The WHCC receives an average of 60–80 referrals per month, with 20 of those specifically related to the population addressed here. Additionally, referrals from the Fontbonne Menopause Clinic within SJHH will be included and recruitment from Hamilton and surrounding areas will take place via advertisement postings and local newspapers. This study will be conducted within the WHCC and has received approval by the Hamilton Integrated Research Ethics Board (HIREB #11152), in accordance with the Declaration of Helsinki.

The consent statement includes comprehensive details of the project such as information regarding the CBT-SC-Peri treatment, assessments, research procedure, potential risks, renumeration, confidentiality, and freedom to withdraw participation. After reading consent and allowed time to ask questions, participants provide their informed consent. Women who do not fulfill initial eligibility are directed to a list of further support services.

#### In Person vs. Virtual Study Visits

As this study will recruit participants from the Greater Hamilton and Toronto Area, and to ensure accessibility for participant's who may be unable to attend in-person visits (i.e., due to long distances, unable to travel), we will provide the option for virtual study visits. The WHCC has the capacity to provide appointments with participants through SJHH's virtual platform, Dovetale, which uses the Zoom Video Communications interface. Zoom provides videotelephony and online chat services through a cloud-based peer-to-peer software platform, used for teleconferencing, telecommuting, distance education, and social relations. Zoom claims to be compliant with the Personal Information Protection and Electronic Documents Act and the Personal Health Information Protection Act. If participants are taking part in a virtual visit for their first study visit, participants will be emailed a copy of the participant information and consent form prior to the first study visit. At the beginning of the first study visit, the interviewer will review the consent form with the participant, via the share screen function on Zoom, providing the opportunity for a question-and-answer period. Following review of the form, participants will be asked to sign and return the consent form, via email, while on Zoom. Once participants have signed and returned the consent form, interviewers will also sign the consent form, and return a copy to the participant via email, before commencing with the first study visit. A section describing virtual visits and the rules for participation are included in the Informed Consent Forms.

#### Telephone Screening and Study Visits

Women who are interested in participating will receive an initial telephone screen to ensure eligibility. Following informed consent, participants deemed eligible will be enrolled into the study and complete their baseline assessment consisting of a structured clinical interview (Mini International Neuropsychiatric Interview for DSM-5 and the Sexual Disorders Module of the Diagnostic and Research Tool for DSM-5) and self-report measures. Upon confirming eligibility at the baseline assessment, participants will be assigned to a treating clinician to undergo four weekly sessions (up to 90 min) of individual CBT-SC-Peri. If a treating clinician is not immediately available, participants will be placed on a waitlist for 4 weeks before starting treatment. Participants who are on the waitlist for treatment will be asked to complete the self-report measures following the waitlist period/before starting treatment. To reduce attrition, participants will be compensated with a $20 gift card following each assessment visit at baseline, post-waitlist (for those in the waitlist group), and post-treatment, in addition to travel reimbursement via the form of a parking pass or bus tickets (if an in-person visit is conducted).

Because of the short duration of the treatment and the sensitive nature of the topic (sexual concerns), there may be an unintended effect of the study namely, no improvement or worsening of symptoms. Should this occur, the research participant will be offered further care by our treatment team within the WHCC, a clinic that is equipped with a multidisciplinary team including psychiatrists, obstetricians-gynecologists, and psychologists.

### Study Measures

The assessment battery includes various measures of sexual concerns, vasomotor symptoms, mood, anxiety, relationship, health, body image, cognitive flexibility, and treatment satisfaction. The primary outcome of this trial, sexual satisfaction, is assessed with the Female Sexual Desire Questionnaire. Please see Table 1 for a list of measures and their associated timing.

**Table 1:** Timing of measurements.

**Procedures and Measures**	**CBT-SC-PERI**		**Waitlist** **+** **CBT-SC-PERI**		
	**Baseline**	**Post-treatment**	**Baseline**	**Post-waitlist**	**Post-treatment**
**Enrolment**					
Consent and screen	X		X		
Allocation	X		X		
**Structured Interviews**					
MINI for DSM-5	X		X		
DART module	X		X		
**Primary Outcomes**					
Female Sexual Function Index	X	X	X	X	X
**Secondary Outcomes**					
Female Sexual Distress Scale-Revised	X	X	X	X	X
Female Sexual Desire Questionnaire	X	X	X	X	X
Hamilton Anxiety Rating Scale	X	X	X	X	X
Hot Flash Related Daily Interference Scale	X	X	X	X	X
Beck Depression Inventory-II	X	X	X	X	X
Couples Satisfaction Index	X	X	X	X	X
36-Item Short-Form Health Survey	X	X	X	X	X
Dresden Body Image Questionnaire	X	X	X	X	X
Cognitive Flexibility Inventory	X	X	X	X	X
**Other factors**					
Demographics Questionnaire	X		X		

*CBT-SC-PERI, Cognitive Behavioral Therapy for Sexual Concerns During Perimenopause; MINI, Mini International Neuropsychiatric Interview; DART, Diagnostic and Assessment Research Tool*.

### Primary Outcome

#### The Female Sexual Function Index

The Female Sexual Function Index (FSFI) (23) is a six-domain measure of female sexual functioning including sexual desire, subjective arousal, lubrication, orgasm, satisfaction, and pain. The FSFI has demonstrated excellent internal consistency (α = 0.82–0.96) and test-retest reliability when used in menopausal women (22–24). A clinical cut-off score of 26 or lower has been suggested to identify significant sexual dysfunction (22). The FSFI is considered as a gold-standard in assessing sexual functioning in women (25).

### Secondary Outcomes

#### Female Sexual Distress Scale-Revised

The Female Sexual Distress Scale-Revised (FSDS-R) (26) is a 13-item self-report questionnaire assessing various aspects of sexual distress, with a clinical cut-off score of 11 indicated sexual distress. The FSDS-R has demonstrated excellent internal consistency (α = 0.86) and high test-retest reliability (26).

#### Female Sexual Desire Questionnaire

The Female Sexual Desire Questionnaire (FSDQ) (27) is a 50-item self-report questionnaire assessing one's experience of and sexual desire for women across six domains: dyadic desire, solitary desire, resistance, positive relationship, sexual self-image, and concern. The FSDQ has demonstrated excellent internal consistency across domains (α = 0.80–0.92) and specifically measures both dyadic and solitary desire, which other questionnaires do not (27).

#### The Greene Climacteric Scale

The Greene Climacteric Scale (GCS) (28) is a self-report questionnaire measuring four menopause-related domains: vasomotor symptoms, depression and anxiety, physical complaints, and sexual concerns. The GCS has demonstrated good internal consistency (α = 0.73–0.90) during the menopausal transition (29).

#### The Hot Flash Related Daily Interference Scale

The Hot Flash Related Daily Interference Scale (HFRDIS) (30) assesses the degree to which vasomotor symptoms interfere with daily life. The HFRDIS has demonstrated excellent internal consistency (α > 0.92–0.96) in the literature (30, 31).

#### The Beck Depression Inventory-II

The Beck Depression Inventory-II (BDI-II) (32) is a self-report questionnaire and one of the most widely used tools for measuring depression. The BDI-II has demonstrated excellent internal consistency across studies (α = 0.83–0.96) and is among the most widely used self-report measures for depression symptom severity (33).

#### The Hamilton Anxiety Scale

The Hamilton Anxiety Scale (HAM-A) (34) is a clinician administered questionnaire developed to quantify the severity of anxiety symptomatology across 14 items assessing psychic (i.e., mental agitation, distress) and somatic (i.e., physical complaints) anxiety. The HAM-A has demonstrated good validity and reliability in both anxiety and depression populations and is considered a gold-standard (35).

#### The Couples Satisfaction Index

The Couples Satisfaction Index (CSI) (36) is a 32-item scale designed to measure one's satisfaction in a relationship. Items are scored on a seven-point Likert scale in which respondents indicate their degree of “happiness, all things considered, of your relationship,” with responses ranging from 0 (extremely unhappy) to 6 (perfect). The CSI has demonstrated excellent internal consistency [α = 0.96–0.98; (36, 37)]. As this questionnaire assesses relationship satisfaction, participants who are not in a relationship during the study will not complete this questionnaire.

#### 36-Item Short-Form Health Survey

The 36-Item Short-Form Health Survey (SF-36) (38) is a 36-item self-administered questionnaire assessing one's perception of overall health and well-being. The SF-36 has demonstrated excellent internal consistency (α = 0.79–0.94) for both total and subscale scores (39, 40).

#### Dresden Body Image Questionnaire

The Dresden Body Image Questionnaire (DBIQ) (41) is a 35-item self-report questionnaire assessing one's feelings of their physical appearance across five subscales: body acceptance, sexual fulfillment, physical contact, vitality, and self-aggrandizement. The DBIQ has demonstrated excellent internal consistency (α = 0.80–0.94) for total and subscale scores (41, 42) and test-retest reliability (41).

#### Cognitive Flexibility Inventory

The Cognitive Flexibility Inventory (CFI) (43) is a 20-item self-report questionnaire that assesses three aspects of cognitive flexibility: tendency to perceive difficult situations as controllable, ability to perceive multiple alternative explanations for life occurrences and human behavior, and ability to generate multiple alternative solutions to difficult situations. The CFI has demonstrated good internal consistency (α = 0.79) and high test-retest reliability (43).

### Other Factors

#### Sociodemographic Questionnaire

Information pertaining to participant's age, ethnicity, marital status, parity, education level, income level, medical history, family psychiatric history, etc., will be collected.

### Cognitive Behavioral Therapy for Sexual Concerns During Perimenopause

Cognitive Behavioral Therapy for Sexual Concerns during Perimenopause (CBT-SC-Peri) will be offered in an individual format. It is a four-session protocol that takes place weekly for up to 90 min in duration. Sessions are administered by one of the study investigators or a trained Ph.D.-level graduate student therapist, supervised by the principal investigator (PI) who is a licensed clinical psychologist. Once participants have completed the four treatment sessions, a post-treatment assessment will be completed within 2-weeks, in which the battery of questionnaires will be re-administered. The content provided during each session includes evidence-based strategies designed to address sexual concerns during perimenopause and are provided in detail in Table 2. As this is an individual CBT protocol, content will be tailored to address the specific problems relevant for each participant. Session components include psychoeducation on sexual concerns during perimenopause, cognitive restructuring strategies, behavioral experiments, and therapeutic exposure. Participants are given materials including worksheets both from our published Cognitive Behavioral Workbook for Menopause (44), as well as worksheets that were developed specifically for this protocol.

**Table 2:** CBT-SC-Peri Session-by-Session Content.

**Session 1**	- Introduction and psychoeducation on the menopausal transition in general (including physiological and hormonal changes and common symptoms associated with menopause). Information on the range of common sexual concerns that women experience throughout the transition (e.g., desire, satisfaction, pain, dryness) will be communicated. - Introduction to the CBT treatment model and role of thoughts and how they contribute to sexual concerns - Introduction to thought monitoring - Homework: reading material and worksheets to record practice
**Session 2**	- Check in and review of homework - Introduction to common cognitive distortions and unhelpful beliefs/expectations related to sexual concerns. Identification of participant's specific cognitive distortions. - Introduction to cognitive strategies for more helpful and balanced thinking (best friend and examine the evidence techniques) - Homework: assigned related reading material and worksheets to record practice with cognitive strategies
**Session 3**	- Check in and review of homework - Review of CBT modal and introduction to the role of problematic behaviors in sexual concerns (e.g., avoidance). - Introduction to behavioral experiments to challenge unhelpful beliefs and to promote more helpful and healthy behaviors to address sexual concerns - Homework: assigned related reading material and worksheets to record behavioral experiments and therapeutic exposures. Continued practice of cognitive strategies
**Session 4**	- Check in and review of homework - Continuation of behavioral experiments and previously learned strategies - Identifying longer-term goals and plan for maintaining gains

### Data Management

Data collected in this study will be recorded both on paper copies (e.g., informed consent form), as well as electronically stored and managed with Research Electronic Data Capture [REDCap; (45)]. REDCap is a secure, meta-driven web-application used to build online databases, developed with the support from the National Center for Research Resources (NCRR) and the National Institutes of Health (NIH). REDCap follows strict regulatory compliance with standards including the Health Insurance Portability and Accountability Act (HIPAA) and the Federal Information Security Management Act (FISMA), in addition to other international standards. Only the informed consent forms will contain the participant's full name, and the consent forms will be stored separately from all other participant documents. After obtaining informed consent, participants will be assigned with a de-identifying code, and only this code will be recorded on study documents. Additionally, the password protected list indicating the codes and participant names will be stored separately from all other study documents to ensure participant confidentiality.

### Statistical Analysis Plan

Treatment response will be determined using multiple measures, as identified in the assessment battery, and distinguished between primary and secondary efficacy analyses. A series of repeated measures analysis of covariance (ANCOVA) will be used to examine condition differences. A two (treatment vs. wait-list) by two (time: pre-treatment, post-treatment) repeated measures ANCOVA will be used to examine differences in both primary and secondary outcomes, specifically sexual satisfaction and desire, body image, partner satisfaction, vasomotor symptoms, as well as depression and anxiety symptoms. Covariates for all analyses will include age, current marital status, medication use at baseline, treatment delivery method, and baseline depression and anxiety severity. If continuous variables do not meet the assumptions of normality, as assessed by the Shapiro-Wilk test, we will use non-parametric methods, with alpha levels set at 0.05.

### Dissemination

Study results will be disseminated through peer-reviewed scientific publications, as well as relevant national and international conferences (e.g., World Congress on Women's Mental Health). Further dissemination in the form of media outlets (e.g., social media platforms), to healthcare professionals (e.g., clinical grand rounds) and to relevant organizations (e.g., North American Menopause Society) will be completed. If results demonstrate the effectiveness of the CBT-SC-Peri protocol, and no adverse consequences that outweigh the benefits are observed, it is anticipated that the use of CBT-SC-Peri will be disseminated widely and implemented into menopausal care. Researchers on the study team (e.g., co-investigators) who have made significant contributions to the design, implementation, and analysis of study data, will be granted authorship for planned and unplanned publications. Further, a lay information package summarizing the study results will be constructed for any participant who expresses interest in receiving the results of the study.

## Discussion

Although cognitive and behavioral therapies for the treatment of menopausal symptoms have received empirical support, sexual concerns within this population are often neglected despite their high prevalence. To our knowledge, this study will be the first to investigate the efficacy of a cognitive-behavioral based treatment protocol aimed at improving sexual concerns experienced during perimenopause. While the intention of this treatment is to primarily target sexual concerns, we also anticipate improvements in vasomotor symptoms, anxiety, depression, and relationship satisfaction. If successful, this study will provide an evidence-based, non-pharmacological treatment for women experiencing untreated sexual concerns during perimenopause. Further, this study could provide the basis for a larger randomized controlled clinical trial to confirm the efficacy of this psychological intervention. As consumer demand increases for alternative treatments for these symptoms during perimenopause, this form of treatment may not only be preferred by some, but necessary for others as hormonal treatments, including controversial testosterone treatment, have adverse risks associated with them. Ultimately, this study has the potential to positively impact a significant proportion of the population, as 28.3% of the Canadian population is comprised of women between the ages of 45-64 years old (46). This study will also have a direct impact on healthcare service delivery by utilizing a manualized psychotherapy approach that would allow training of new health professionals, thereby increasing the treatment's availability to consumers.

## Ethics Statement

This study, involving human participants, was reviewed and approved by the Hamilton Integrated Research Ethics Board (HIREB). The patients/participants win this study have/will provide their written informed consent to participate in this study.

## Author Contributions

SG is the PI of this study and she contributed to the conceptualization, methodology, project administration, writing, and editing. MF is a co-investigator of this study and she contributed to the methodology, project administration, writing, and editing. All authors contributed to the article and approved the submitted version.

## Funding

SG and MF are the recipients of the Women's Health Clinical Mentorship Grant (#433269) from the Canadian Institute of Health Research (CIHR) grant in support of this project.

## Conflict of Interest

The authors declare that the research was conducted in the absence of any commercial or financial relationships that could be construed as a potential conflict of interest.

## Publisher's Note

All claims expressed in this article are solely those of the authors and do not necessarily represent those of their affiliated organizations, or those of the publisher, the editors and the reviewers. Any product that may be evaluated in this article, or claim that may be made by its manufacturer, is not guaranteed or endorsed by the publisher.
